# From Recognition to Diagnosis: Caregiver Response, Help-Seeking Pathways, and Access-Related Factors Associated with Autism Diagnostic Delay in Jordan

**DOI:** 10.3390/children13091228

**Published:** 2026-09-11

**Authors:** Hana Taha, Mohammad AlAhmad, Zaid Altawil, Abdalrahman Albakri, Mohammad Alshamasneh, Omar Daas, Amira Masri, Laila Tutunji, Linus Jönsson

**Affiliations:** 1 Department of Family and Community Medicine, School of Medicine, The University of Jordan, Amman 11942, Jordan; mhm0208252@ju.edu.jo (M.A.); zyd0214656@ju.edu.jo (Z.A.); abd0206202@ju.edu.jo (A.A.); mhm0217606@ju.edu.jo (M.A.); omardaas2002@gmail.com (O.D.); 2 Department of Neurobiology, Care Sciences and Society, Karolinska Institutet, 171 77 Stockholm, Sweden; linus.jonsson@ki.se; 3 Department of Pediatrics, Division of Child Neurology, School of Medicine, The University of Jordan, Amman 11942, Jordan; amasri@ju.edu.jo; 4 Section of Pediatric Cardiology, Department of Pediatrics, School of Medicine, The University of Jordan, Amman 11942, Jordan; la.tutunji@ju.edu.jo

**Keywords:** autism spectrum disorder, diagnostic delay, caregivers, help-seeking behavior, healthcare access, Jordan

## Abstract

**Highlights:**

**What are the main findings?**

- Caregivers noticed first concerns at a median age of 2.0 years. However, 60.7% were in the “6 Months to 1 Year” diagnostic delay category or longer (46.2% in the “1–2 Years” category or longer; 24.0% in the “More than 2 Years” category). Longer delay was associated primarily with post-recognition pathway factors rather than background characteristics.
- Longer diagnostic delay was independently associated with caregivers who reported that early signs had initially not been acted upon because they were interpreted as part of normal development. It was also associated with first contact via a speech/learning center or other service rather than a pediatrician, and with caregiver-reported previous professional reassurance that the child did not have ASD. Definite appointment difficulty showed a significant Yes-versus-No contrast, although the appointment difficulty variable was not statistically significant in the global test.

**What are the implications of the main findings?**

- Within the post-recognition interval, caregiver response, help-seeking route, access, and professional response were associated with diagnostic delay. The models do not determine where delay accumulated within individual pathway stages.
- These findings support clearer referral pathways, explicit follow-up after professional reassurance, and timely access to appropriate developmental assessment as potential priorities for shortening the interval between first concern and diagnosis.

**Abstract:**

**Background:** Autism spectrum disorder (ASD) is often diagnosed well after developmental concerns first emerge, and evidence on factors associated with the duration of the recognition-to-diagnosis pathway remains limited in the Middle East. This study aimed to identify factors associated with the overall recognition-to-diagnosis interval and potential areas for earlier recognition, referral, and access to appropriate assessment. **Methods:** This multisite cross-sectional survey of 384 caregivers of children with confirmed ASD was conducted across Jordanian governorates. Diagnostic timing was known for 338 participants. Five sequential nested ordinal logistic regression models were fitted on a common complete-case sample (N = 299), successively adding background characteristics, recognition, caregiver response, help-seeking route, and access/professional response variables. Robustness was assessed via grouping-specific binary models, multiple imputation, and bootstrap resampling. **Results:** Caregivers reported first concerns at a median age of 2.0 years. Among those with known diagnostic timing, 60.7% were in the “6 Months to 1 Year” delay category or longer, 46.2% were in the “1–2 Years” category or longer, and 24.0% were in the “More than 2 Years” category. The background characteristics model showed limited explanatory capacity (Nagelkerke R^2^ = 0.021), and adding recognition variables did not improve model fit (*p* = 0.562). Fit improved significantly with caregiver response (*p* = 0.001), help-seeking route (*p* = 0.008), and access/professional response (*p* < 0.001). The final model reached a Nagelkerke R^2^ = 0.178, indicating modest overall explanatory capacity. Longer diagnostic delay was independently associated with caregivers who reported that early signs had initially not been acted upon because they were interpreted as part of normal development (AOR = 2.21). It was also associated with first contact via a speech/learning center (AOR = 2.27) or other service (AOR = 2.72) rather than a pediatrician. Caregiver-reported previous professional reassurance that the child did not have ASD was also associated with longer delay (AOR = 2.18). Professional reassurance was the most consistent correlate across sensitivity analyses. Definite appointment difficulty showed a significant Yes-versus-No contrast (AOR = 1.81), although the appointment difficulty variable was not statistically significant in the global test. Sociodemographic factors showed no independent association. Among 11 exploratory barriers, only prior misdiagnosis survived multiplicity correction (AOR = 2.21). **Conclusions:** In this Jordanian cohort, the length of the recognition-to-diagnosis interval was associated with factors operating after developmental concerns were first recognized, rather than with the timing or breadth of recognition itself. Caregiver response, entry route into care, and professional response emerged as potentially important pathway markers. However, the modest explanatory capacity of the final model indicates that substantial variability in diagnostic delay remains unaccounted for by the measured variables. These findings support provider- and system-level measures, including clearer referral pathways, explicit follow-up when reassurance is provided, improved appointment access, and expanded diagnostic capacity, complemented by caregiver-facing information and support.

## 1. Background

Autism spectrum disorder (ASD) is a neurodevelopmental condition characterized by differences in social communication and interaction, together with restricted or repetitive patterns of behavior, interests, or activities. ASD affects approximately 1% of children worldwide, although reported prevalence varies across countries and settings. Differences in awareness, case identification, diagnostic practices, and access to healthcare services may contribute to variation in reported prevalence [1,2].

Timely identification of ASD matters for more than access to intervention. It also facilitates access to appropriate supports, accommodations, information, and services and helps families understand and engage in care. Evidence-based recommendations emphasize the importance of early intervention for young children with suspected or confirmed ASD and highlight the central role of parents and caregivers in intervention [3]. Nevertheless, ASD is often diagnosed considerably later than developmental differences are first recognized. International studies have reported mean ages at diagnosis ranging from approximately 38 to 120 months, while a systematic review and meta-analysis of studies published between 2012 and 2019 estimated the average age at diagnosis at approximately 60 months [4,5].

The pathway from the first recognition of developmental concerns to a confirmed ASD diagnosis is influenced by factors beyond the child’s clinical presentation. Caregivers are often among the first people to recognize differences in speech, social interaction, eye contact, play, or behavior; however, recognition of these differences does not necessarily lead immediately to professional assessment. Parents may have difficulty interpreting developmental differences or may initially consider them part of normal development or a temporary problem. Their responses can be influenced by autism knowledge, cultural expectations, stigma, advice from family members, and previous parenting experience [6,7,8,9]. Research has also shown that parental concerns may not always be acted upon promptly, particularly when parents encounter uncertainty or receive responses that do not immediately lead to further assessment [7]. Caregiver interpretation of early signs does not occur in isolation. Without routine developmental surveillance or clear guidance on when and how to seek assessment, caregivers may reasonably monitor developmental differences before seeking help. This should not necessarily be interpreted as a lack of knowledge or motivation.

Professional responses represent another important stage in the diagnostic pathway. Some children are referred promptly for specialist assessment. Others may be reassured that their development is normal, advised to wait, or referred for evaluation of an individual difficulty such as speech delay rather than for a broader developmental assessment [6,7,10]. In particular, qualitative research has documented experiences in which parents who had actively raised concerns were reassured by healthcare professionals, potentially contributing to delays in obtaining an ASD diagnosis [6,10]. Primary care and other frontline professionals therefore have an important role in recognizing developmental concerns and facilitating appropriate referral and assessment [9,11].

Even when families seek professional help, access to appropriate diagnostic services can create additional delays. Families may encounter multiple professionals, referral procedures, waiting lists, and lengthy assessment processes before reaching an appropriate diagnostic service. In a survey of Canadian pediatricians, reported referral-to-diagnosis waiting times ranged from 2 to 24 months, with a median of 7 months [12]. System-level factors contributing to delays include shortages of specialist providers, variation in diagnostic practices, prolonged assessment procedures, and limited availability of appropriate services [9,12]. These barriers may be particularly important in rural and underserved communities, where geographic distance, limited resources, staffing shortages, and difficulties navigating complex service systems can further impede access to diagnosis [8,13,14].

The challenges associated with ASD diagnosis may be especially relevant in low- and middle-income countries (LMICs), where barriers can occur across the stages of detection, diagnosis, referral, and access to intervention. Reviews of autism services in LMICs have identified difficulties related to limited specialist capacity, health-system constraints, social and family factors, and restricted access to appropriate services [15,16]. Research from underserved populations has additionally highlighted the role of cultural and systemic factors in shaping families’ experiences of seeking an autism diagnosis [6,8,14]. This evidence suggests that diagnostic delay should be considered not only as a consequence of the child’s clinical presentation but also as a process involving caregivers, professionals, referral systems, and service availability.

Evidence from Jordan indicates that diagnostic delay and difficulties in accessing autism-related services are also important concerns. In a review of 84 children diagnosed with autism at Jordan University Hospital, the mean age at diagnosis was 3.8 years, with ages ranging from 14 months to 9 years [17]. More recent qualitative research involving Jordanian parents identified concerns related to the duration and organization of the diagnostic process, communication with professionals, the information and support provided to families, and the use of diagnostic tools. The study also highlighted the influence of societal stigma within the local cultural context [18]. Access to autism intervention services remains another challenge in Jordan. In a study of 274 children with ASD, the most frequently reported barriers to receiving rehabilitation services were financial cost and transportation [19].

Although these studies provide important evidence about ASD diagnosis and service access in Jordan, they have largely examined particular components of the diagnostic experience rather than the entire pathway from initial recognition of developmental concerns to confirmed diagnosis. International research has similarly examined different stages of this process, including age at diagnosis, parental concerns, professional responses, waiting times, and barriers to accessing services [4,10,12,14]. Consequently, less is known about how caregiver recognition and interpretation of early signs, help-seeking behavior, professional responses, specialist evaluation, referral processes, and service accessibility interact across the diagnostic journey.

Examining this pathway as a whole may help identify caregiver-, provider-, referral-, and access-related factors associated with the overall recognition-to-diagnosis interval. This is particularly relevant in Jordan, where existing evidence has identified concerns related to diagnostic experiences, professional interactions, and access to autism services [17,18,19,20,21]. The present study therefore examines the pathway from first recognition of developmental concerns to confirmed ASD diagnosis among caregivers in Jordan. It aims to identify factors associated with the duration of this interval and potential areas for earlier recognition, referral, and access to appropriate assessment. The design does not measure the duration of individual stages or determine where delay accumulated within the pathway.

## 2. Materials and Methods

### 2.1. Study Design and Setting

This multisite cross-sectional caregiver survey examined the pathway from first developmental concern to confirmed autism spectrum disorder (ASD) diagnosis in Jordan. Data were collected between January and July 2026 through autism centers, special-education settings, clinical services, and caregiver and community networks across multiple governorates. Recruitment aimed to include families from different geographic and socioeconomic backgrounds, including urban, rural, and Bedouin communities. Because part of the sample was recruited through an open electronic link, a conventional response rate could not be calculated.

### 2.2. Study Population, Eligibility, and Sample Flow

The study population comprised caregivers of sons or daughters with a professionally confirmed diagnosis of ASD. ASD status had been established before study enrollment and was not inferred from questionnaire responses. Eligible children had a professionally confirmed ASD diagnosis established before study enrollment by a treating physician or specialist, as reflected in hospital, charity, or service-organization ASD records. Diagnostic status was not independently re-verified by the study team against medical documentation or through a study-administered standardized instrument (e.g., ADOS-2, ADI-R). Instead, it relied on prior clinical diagnosis and current registry status. The diagnosing specialty or credential was not uniformly recorded across sites. Eligible participants were caregivers who were able to complete the Arabic-language questionnaire and provide informed consent; no age restriction was applied to the son or daughter with ASD. A total of 389 responses were submitted. Five responses were excluded during eligibility screening, leaving a final eligible cohort of 384 caregivers. Diagnostic timing was available for 338 participants and unavailable for 46. Among the 338 participants with known diagnostic timing, 299 had complete data for all variables in the primary M1–M5 analysis. The remaining 39 had one or more unavailable covariate values, including explicit unknown responses treated as unavailable in the primary model. Retaining explicit unknown predictor responses as separate categories increased the usable sample to 331 in the unknown-inclusive sensitivity model. The remaining seven participants lacked other required covariate information or included the single non-estimable unknown first contact response. Multiple imputation addressed covariate incompleteness among all 338 participants with known diagnostic timing. The diagnostic delay outcome itself was not imputed. The 46 participants with unknown diagnostic timing were therefore distinct from the 39 participants who did not enter the primary complete-case model.

### 2.3. Sampling Strategy and Sample Size

A nonprobability convenience sampling strategy was used. The target sample size for the broader cross-sectional survey was approximately 384 participants, based on the conventional Cochran approach using a conservative expected proportion of 50%, a 95% confidence level, and a 5% margin of error. The final eligible cohort met this target. This calculation was used to guide descriptive precision for the broader survey. Regression analyses used the eligible analytic samples defined by outcome and covariate availability.

### 2.4. Data Collection Procedure

A total of 389 caregiver responses were submitted. Recruitment was conducted across multiple governorates through caregiver and community networks. Participants were recruited through centers affiliated with the Ministry of Social Development (n = 160; 115 online and 45 face-to-face) and autism centers or special-education settings (n = 145; 110 online and 35 face-to-face). Clinical recruitment included the University of Jordan Hospital (n = 54; 44 online and 10 face-to-face) and the Hussein Center for Early Diagnosis of Disabilities (n = 30; 15 online and 15 face-to-face). Overall, 284 of the 389 submitted responses (73.0%) were completed online and 105 (27.0%) face-to-face. Data were collected using a structured Arabic-language caregiver questionnaire. Both formats used the same wording and response options. The broader instrument was reviewed for content relevance and cultural appropriateness and was piloted before full data collection. Participation was voluntary, and responses were handled confidentially.

### 2.5. Study Instrument and Recognition-to-Diagnosis Framework

For the present paper, analyses were restricted to variables directly relevant to the pathway from recognition of developmental concerns to confirmed ASD diagnosis. Knowledge and attitude measures from the broader survey were reserved for a separate companion analysis and were not included in the present pathway models.

Stage 1—Background characteristics. Variables included age and sex of the son or daughter with ASD, maternal education, monthly household income, and place of residence. Residence was represented in the regression models as urban versus non-urban.

Stage 2—Recognition. Recognition variables included age at first developmental concern and reported symptom-domain count. Age at first developmental concern was available for 381 of 384 participants. Six symptom domains were assessed: speech delay, difficulty with eye contact, behavioral problems, difficulty with social interaction, repetitive behaviors, and cognitive or developmental delay. These domains represented a pragmatic set of caregiver-recognizable developmental concerns relevant to ASD recognition, spanning social-communication and repetitive-behavior features as well as commonly reported developmental concerns. They were not derived from a validated symptom inventory and were not intended to provide an exhaustive assessment of ASD-related symptoms. The symptom-domain count, representing the number of these domains reported for each individual, served as a summary measure of symptom breadth in the primary analysis.

Stage 3—Caregiver response. Caregiver response was assessed by asking whether early signs had initially not been acted upon because they were interpreted as part of normal development (recorded in the dataset as “Ignored Early Signs as Normal Phase”). Responses were categorized as No, Maybe, or Yes. This item combines behavior (not acting) and interpretation (viewing signs as normal) in a single measure and is not a validated measure of caregiver knowledge, stigma, or motivation.

Stage 4—Help-seeking pathway. The first professional or service consulted after recognition of developmental concerns was categorized as a pediatrician, general practitioner, autism center, speech or learning center, or other service. The “other service” category (n = 33) was heterogeneous. It included neurologist or pediatric neurologist contacts (n = 8), nonspecified physicians (n = 6), diagnostic/disability or intelligence-assessment centers (n = 7), hospital/emergency or government-facility contacts (n = 4), mixed or multiple-service responses that could not be assigned to a single first contact route (n = 5), and three additional individual responses. Prior specialist evaluation before diagnostic confirmation was also recorded. The number of evaluations before diagnosis was described as a pathway characteristic but was not included in the primary sequential model because 26.6% of participants could not recall this information.

Stage 5—Access and professional response. Access and professional response variables included difficulty obtaining appointments, categorized as No, Sometimes, Yes, or unknown, and previous professional reassurance that the individual did not have ASD, categorized as Yes, No, or unknown. Because appointment difficulty and previous reassurance arise during the interval between first concern and confirmed diagnosis, they were treated as markers of the diagnostic pathway and interpreted non-causally.

Caregiver-attributed reasons for delay were examined separately as exploratory barrier variables and were not entered into the primary sequential pathway model.

### 2.6. Outcome Variable

The primary outcome was caregiver-reported diagnostic delay, defined as the interval between first recognized developmental concern and confirmed ASD diagnosis. The questionnaire response options were “Less than 3 Months,” “3–6 Months,” “6 Months to 1 Year,” “1–2 Years,” “More than 2 Years,” and “Do not remember.” The five known timing categories were retained in this original order as the primary ordinal outcome. For cumulative analyses, categories were grouped according to the original response options. The “6 Months to 1 Year or longer” analysis combined “6 Months to 1 Year,” “1–2 Years,” and “More than 2 Years.” The “1–2 Years or longer” analysis combined “1–2 Years” and “More than 2 Years,” while the >2-year analysis included only “More than 2 Years.” Because exact boundary-month assignment was not collected, these labels refer to response-category groupings rather than exact dichotomous time cutoffs. Participants selecting “Do not remember” were treated as having unavailable outcome timing and were excluded from analyses requiring assignment to a delay category.

### 2.7. Descriptive Analysis

Continuous variables were summarized using means and standard deviations (SDs) or medians and interquartile ranges (IQRs), as appropriate. Age at first developmental concern was right-skewed and was therefore summarized using the median and IQR. Categorical variables were summarized using frequencies and percentages. Denominators were based on available observations and are specified where they differed from the full cohort.

### 2.8. Primary Ordinal Model and Sequential Framework

The primary analysis used ordinal logistic regression with the five-level ordered diagnostic delay outcome. Ordinal regression was selected because diagnostic delay was recorded in ordered caregiver-reported categories of unequal width rather than as exact event times. Adjusted odds ratios (AORs) greater than 1 indicate higher odds of belonging to a longer diagnostic delay category.

To examine the staged recognition-to-diagnosis framework, five sequential nested models were fitted using the same complete-case sample (N = 299). This ensured that changes in model fit reflected added pathway blocks rather than changes in participants. Model 1 included background characteristics: age and sex of the individual with ASD, maternal education, household income, and urban residence. Model 2 added age at first concern and symptom-domain count. Model 3 added caregiver response to early signs. Model 4 added first contact route and prior specialist evaluation. Model 5 added appointment difficulty and previous professional reassurance. Pediatrician was the reference category for first contact route; No was the reference category for caregiver response and appointment difficulty. Maternal education and household income were modeled as ordered trends. A sensitivity analysis compared this coding with full categorical parameterization.

### 2.9. Model Evaluation and Diagnostics

Model progression was evaluated using likelihood-ratio (LR) tests between consecutive models, Akaike information criterion (AIC), Bayesian information criterion (BIC), and McFadden and Nagelkerke pseudo-R^2^ measures. In the fully adjusted model, global LR tests were also used to evaluate the caregiver response, first contact route, and appointment difficulty blocks. The proportional-odds assumption was assessed using an omnibus stacked-score Wald diagnostic together with predictor-specific local diagnostics.

### 2.10. Sensitivity and Robustness Analyses

Binary logistic regression models used the same covariate structure as the fully adjusted model. They were fitted for the “6 Months to 1 Year or longer” and “1–2 Years or longer” response-category groupings and for the exploratory > 2-year outcome. These grouping-specific models were used to evaluate whether associations differed across the cumulative response-category groupings and to aid interpretation of any departure from proportional odds. Predictor-specific diagnostics indicated localized departures from proportional odds. We therefore fitted a partial proportional-odds generalized ordered logistic model using the same complete-case sample (N = 299) and covariate structure as Model 5. Age at first concern and the Maybe-versus-No caregiver response contrast were allowed to vary across the four cumulative response-category groupings, while proportional effects were retained for the remaining predictors. Missing covariate data were examined in a sensitivity analysis using 20 imputed datasets among the 338 participants with known diagnostic timing. The diagnostic delay outcome was not imputed, and pooled estimates were combined using Rubin’s rules. Coefficient stability for the highlighted coefficient-level findings was further assessed using 200 bootstrap resamples of the fully adjusted ordinal model.

We also examined whether the aggregate symptom-domain count obscured associations with specific symptom presentations. The full M1–M5 sequence was repeated in the same complete-case sample (N = 299), replacing the symptom-domain count with six individual-symptom indicators: speech delay, eye contact difficulty, behavioral problems, social interaction difficulty, repetitive behaviors, and cognitive/developmental delay. The aggregate count and its component indicators were not entered simultaneously. The fully adjusted individual-symptom specification was compared with the primary symptom-domain count specification using a likelihood-ratio test.

### 2.11. Exploratory Analyses and Missing-Outcome Assessment

Caregiver-attributed barriers were examined in separate adjusted ordinal logistic regression models that included age and sex of the individual with ASD, maternal education, household income, and urban residence. Benjamini–Hochberg correction was applied across the 11 barrier analyses. Participants with known and unknown diagnostic timing were compared to evaluate potential differences related to outcome nonresponse. Age was compared using the Mann–Whitney U test, and categorical variables were compared using Pearson’s chi-square or Fisher’s exact test, as appropriate. No multiplicity correction was applied to individual coefficients in the primary sequential models. Interpretation emphasized block-level LR tests, effect estimates with 95% confidence intervals, and consistency across sensitivity analyses. All statistical tests were two-tailed, and *p* < 0.05 was considered statistically significant. Analyses were conducted using R version 4.3 and Python version 3.11.

Explicit “Unknown/Do not remember” responses were analyzed separately. Diagnostic timing availability was modeled as known versus unknown, rather than treating “Do not remember” as an additional ordinal delay category. The known-versus-unknown timing model included 379 participants with the required covariates. The fully adjusted ordinal model was also repeated, retaining explicit unknown responses as separate categories for prior specialist evaluation, appointment difficulty, and previous professional reassurance.

### 2.12. Ethical Considerations

Ethical approval was obtained from the Scientific Research Committee of the Faculty of Medicine at the University of Jordan (Approval No. 7849/2025/67), the Institutional Review Board of Jordan University Hospital (Approval No. 10/2025/32922), the Institutional Ethics Committee of the Jordanian Ministry of Health (Approval No. MOH/REC/2025/49), and the Research Approval Committee of the Jordanian Ministry of Social Development (Approval No. SW/3/22331). Informed consent was obtained before questionnaire completion. Participation was voluntary, and confidentiality and anonymity were maintained throughout the study.

## 3. Results

### 3.1. Participant Flow and Characteristics

Among 389 submitted responses, five were excluded during eligibility screening, leaving 384 eligible caregivers. Diagnostic timing was known for 338 participants and unknown for 46. The common complete-case sample for the five sequential ordinal models comprised 299 participants.

The mean age of sons and daughters with ASD was 10.0 ± 5.0 years, with a median age of 9 years (IQR: 6–13). Most were male (78.1%). Nearly half attended special-needs education centers (48.7%), whereas approximately one-quarter did not attend school (25.8%). Most families reported a monthly household income of 600 JOD or less (83.3%), and 70.1% lived in cities. Health insurance coverage was reported for 82.3%, and 7.0% reported a confirmed family history of ASD. Participant characteristics are summarized in Table 1.

### 3.2. Recognition, Caregiver Response, and the Diagnostic Pathway

Caregivers reported first developmental concerns at a median age of 2.0 years (IQR: 1.5–3.0; N = 381), with speech delay the most frequently reported concern (83.3%). Early signs were initially not acted upon because they were interpreted as part of normal development by 47.7% of caregivers. Pediatricians were the most common first point of contact (35.5%), and 38.0% of caregivers reported having previously received professional reassurance that the individual did not have ASD. Among the 338 participants with known diagnostic timing, 60.7% were in the “6 Months to 1 Year” category or longer, 46.2% were in the “1–2 Years” category or longer, and 24.0% were in the “More than 2 Years” category. Full pathway characteristics and diagnostic delay distributions are presented in Table 2.

### 3.3. Sequential Nested Models of Diagnostic Delay

Background characteristics alone explained little variation in diagnostic delay. Adding recognition variables did not significantly improve model fit (M2 vs. M1, *p* = 0.562). Model fit improved after caregiver response was added (M3 vs. M2, *p* = 0.001) and improved further after help-seeking route variables were introduced (M4 vs. M3, *p* = 0.008). It improved again after access and professional response variables were added (M5 vs. M4, *p* < 0.001). Nagelkerke pseudo-R^2^ increased from 0.021 in M1 to 0.178 in M5. AIC was lowest for M5, whereas BIC favored the more parsimonious M1 model. Full categorical coding of maternal education and household income did not improve fit compared with ordered-trend coding (LR *p* = 0.888).

Given the modest departure from the proportional-odds assumption, the ordinal estimates should be interpreted as average associations across the ordered delay categories rather than uniform effects across all cumulative groupings. The primary ordinal estimates are presented in Table 3, with grouping-specific estimates provided in Table 4. In the fully adjusted model, caregivers who reported that early signs had initially not been acted upon because they were interpreted as part of normal development had more than twice the odds of longer diagnostic delay (AOR = 2.21, 95% CI: 1.38–3.54, *p* = 0.001). First contact through a speech or learning center (AOR = 2.27, 95% CI: 1.20–4.27, *p* = 0.011) and through other services (AOR = 2.72, 95% CI: 1.22–6.09, *p* = 0.015) were also associated with longer diagnostic delay compared with pediatrician first contact. Definite appointment difficulty was associated with longer diagnostic delay compared with no difficulty (AOR = 1.81, 95% CI: 1.08–3.02, *p* = 0.023). However, the appointment difficulty variable as a whole was not statistically significant in the global test (*p* = 0.070). Caregivers’ report of having previously received professional reassurance that the individual did not have ASD was associated with longer diagnostic delay (AOR = 2.18, 95% CI: 1.40–3.38, *p* < 0.001). The final model had a Nagelkerke pseudo-R^2^ of 0.178, indicating modest explanatory capacity and suggesting that substantial variability in diagnostic delay remained unaccounted for by the measured variables.

Age and sex of the individual with ASD, maternal education, household income, residence, age at first concern, symptom-domain count, the Maybe-versus-No caregiver response contrast, general-practitioner first contact, autism-center first contact, and prior specialist evaluation were not independently associated with longer delay. Global block tests supported the contribution of caregiver response (*p* = 0.004) and first contact route (*p* = 0.022). The overall appointment difficulty block did not reach conventional statistical significance (*p* = 0.070), despite the significant Yes-versus-No contrast. Complete coefficient estimates across M1–M5 are provided in Supplementary Table S3.

In the individual-symptom sensitivity analysis, speech delay was associated with longer diagnostic delay in M2 (AOR = 1.95, 95% CI: 1.02–3.72, *p* = 0.042), although the recognition block as a whole did not significantly improve model fit over M1 (LR *p* = 0.425). The association with speech delay attenuated after subsequent pathway variables were added and was not statistically significant in M5 (AOR = 1.51, 95% CI: 0.77–2.95, *p* = 0.229). None of the six individual-symptom domains was independently associated with delay in the fully adjusted model. The five highlighted coefficient-level associations remained largely unchanged. These included caregiver response Yes versus No (AOR = 2.25, *p* < 0.001), speech/learning center first contact (AOR = 2.20, *p* = 0.015), and other service first contact (AOR = 2.71, *p* = 0.017). The appointment difficulty Yes-versus-No contrast (AOR = 1.76, *p* = 0.031) and previous professional reassurance (AOR = 2.20, *p* < 0.001) also remained significant. The individual-symptom specification did not significantly improve the fully adjusted model compared with the symptom-domain count specification (LR χ^2^ = 4.20, df = 5, *p* = 0.521; Supplementary Table S5).

### 3.4. Robustness and Model Diagnostics

The omnibus proportional-odds diagnostic indicated a modest departure from the proportional-odds assumption (χ^2^ = 69.61, df = 51, *p* = 0.043). Predictor-specific diagnostics suggested that this departure was concentrated primarily in age at first concern (*p* = 0.011) and the Maybe-versus-No caregiver response contrast (*p* = 0.028), with borderline variation across cumulative response-category groupings for speech or learning first contact (*p* = 0.074). A partial proportional-odds generalized ordered logistic model was therefore fitted as a supplementary robustness analysis. Age at first concern and the Maybe-versus-No caregiver response contrast were allowed to vary across cumulative response-category groupings, while proportional effects were retained for the remaining predictors. The partial proportional-odds model improved fit relative to the fully constrained proportional-odds model (LR χ^2^ = 16.15, df = 6, *p* = 0.013). Importantly, the five highlighted coefficient-level associations remained statistically significant and were similar in magnitude. These included caregiver response Yes versus No (AOR = 2.34, 95% CI: 1.45–3.77, *p* < 0.001), speech or learning first contact (AOR = 2.34, 95% CI: 1.23–4.43, *p* = 0.009), and other service first contact (AOR = 3.05, 95% CI: 1.35–6.91, *p* = 0.007). The appointment difficulty Yes-versus-No contrast (AOR = 1.82, 95% CI: 1.08–3.06, *p* = 0.023) and previous professional reassurance (AOR = 2.16, 95% CI: 1.39–3.37, *p* < 0.001) also remained significant. Age at first concern was not statistically significant in any individual cumulative grouping. The Maybe-versus-No caregiver response contrast increased across cumulative groupings and was statistically significant only for the >2-year grouping (AOR = 2.61, 95% CI: 1.06–6.41, *p* = 0.036). These findings indicate that the observed non-proportionality was localized and did not materially alter the main coefficient-level findings; full estimates are provided in Supplementary Table S4.

The highlighted coefficient-level findings were broadly consistent across alternative outcome definitions and the missing-data sensitivity analysis, although the strength of association differed by outcome grouping. Early signs initially not acted upon because they were interpreted as part of normal development were associated with the “6 Months to 1 Year or longer” grouping and with the exploratory > 2-year outcome. They were not specifically associated with the “1–2 Years or longer” grouping. The Maybe-versus-No caregiver response contrast was significant only for the exploratory > 2-year outcome (AOR = 3.58, 95% CI: 1.30–9.85). It was not significant in the ordinal, “6 Months to 1 Year or longer,” “1–2 Years or longer,” or multiple imputation models, so this isolated finding should be interpreted cautiously. Speech or learning first contact and other first contact routes showed their strongest associations with the “1–2 Years or longer” grouping. Definite appointment difficulty was associated with the “1–2 Years or longer” and >2-year outcomes. Its association with the “6 Months to 1 Year or longer” outcome was borderline (*p* = 0.050). Previous professional reassurance was associated with all three binary delay outcomes.

In the multiple imputation analysis of all 338 participants with known diagnostic timing, all five highlighted coefficient-level associations remained statistically significant. These included caregiver response (AOR = 1.96, 95% CI: 1.26–3.05), speech or learning first contact (AOR = 2.08, 95% CI: 1.14–3.79), and other first contact routes (AOR = 3.00, 95% CI: 1.42–6.34). The appointment difficulty Yes-versus-No contrast (AOR = 2.10, 95% CI: 1.28–3.45) and previous professional reassurance (AOR = 1.92, 95% CI: 1.26–2.94) also remained significant. These estimates were materially similar to the complete-case results, reducing concern that excluding participants with unavailable covariate values under the primary complete-case coding materially drove the main findings. Across 200 bootstrap resamples, the estimated direction was positive in 99.5% of resamples for caregiver response and 99.5% for speech or learning first contact. The corresponding proportions were 98.0% for other first contact routes, 98.5% for the appointment difficulty Yes-versus-No contrast, and 100% for previous professional reassurance.

### 3.5. Exploratory and Supplementary Analyses

In the exploratory caregiver-attributed barrier analysis, previous misdiagnosis was the only one of the 11 barriers that remained associated with longer diagnostic delay after Benjamini–Hochberg correction (adjusted OR = 2.21, 95% CI: 1.35–3.61, adjusted *p* = 0.017; Supplementary Table S1). Before multiplicity correction, lack of knowledge/awareness was nominally associated with longer diagnostic delay, while difficulty accessing healthcare showed a borderline association; neither remained statistically significant after Benjamini–Hochberg correction.

Participants with unknown diagnostic timing differed from those with known timing in maternal education (*p* = 0.007), caregiver response to early signs (*p* = 0.004), appointment difficulty responses (*p* < 0.001), and professional reassurance responses (*p* = 0.003). Age, sex, household income, residence, and first contact route did not differ significantly between timing-status groups. These findings indicate that missing diagnostic timing data may not have been completely random (Supplementary Table S2).

Diagnostic timing availability was also modeled as known versus unknown. Recall-related unknown responses were associated with diagnostic timing availability. Inability to recall the number of evaluations was associated with greater odds of unknown diagnostic timing (AOR = 2.70, 95% CI: 1.26–5.78, *p* = 0.011). An unknown response regarding previous professional reassurance was also associated with greater odds of unknown timing (AOR = 3.96, 95% CI: 1.19–13.23, *p* = 0.025). These associations were interpreted as reporting/recall patterns rather than causal effects. The fully adjusted ordinal model was repeated with explicit unknown predictor categories, increasing the analytic sample from N = 299 to N = 331. The five highlighted coefficient-level associations remained materially unchanged. These included caregiver response Yes versus No (AOR = 2.14, *p* = 0.001), speech/learning center first contact (AOR = 2.29, *p* = 0.008), and other service first contact (AOR = 2.55, *p* = 0.015). The appointment difficulty Yes-versus-No contrast (AOR = 1.79, *p* = 0.022) and previous professional reassurance (AOR = 2.17, *p* < 0.001) also remained significant. Unknown appointment difficulty was also associated with longer reported delay (AOR = 4.41, 95% CI: 1.17–16.69, *p* = 0.029), although the estimate was imprecise because of the small subgroup (Supplementary Table S6).

## 4. Discussion

Sequential nested modeling on a common sample of 299 participants showed that the background characteristics model had limited explanatory capacity and that adding recognition variables did not improve model fit (*p* = 0.562). Model fit improved after caregiver response variables were introduced (*p* = 0.001), improved further with the addition of help-seeking route (*p* = 0.008), and improved again after access and professional response variables were added (*p* < 0.001). Nagelkerke pseudo-R^2^ increased from 0.021 to 0.178. These sequential improvements indicate that caregiver response, first contact route, and access/professional response variables provided additional information about variation in the total diagnostic delay interval. However, the models do not establish how much of that interval was spent at any specific stage of the diagnostic pathway. The final model nevertheless retained only modest explanatory capacity, suggesting that substantial variability in diagnostic delay was not captured by the measured variables. Longer delay was independently associated with caregivers who reported that early signs had initially not been acted upon because they were interpreted as part of normal development. It was also associated with first contact through a speech or learning center or other service and with caregiver-reported previous professional reassurance that the individual did not have autism. Definite appointment difficulty showed a significant Yes-versus-No contrast. However, the appointment difficulty variable was not statistically significant in the global test and should therefore be interpreted cautiously.

The magnitude of delay observed in this cohort lies toward the upper end of the published range while remaining within internationally reported patterns. A retrospective cohort of 480 Chinese children reported a median delay of 9.58 months, with 25% of cases exceeding 24.59 months [22]. A caregiver survey spanning six Latin American and Caribbean countries identified first concerns at a mean of 22 months and diagnosis approximately 24 months thereafter [23]. A second Chinese sample reported a mean concern-to-confirmation interval of 10.93 months, with 86.24% of children diagnosed after 24 months of age [24]. Among more than 1000 caregivers in the United Kingdom, approximately 1 year elapsed before help was sought and a further 3.6 years before diagnostic confirmation [25]. An Australian investigation recorded a mean age of 2 years 8 months at first professional consultation [26]. The Jordanian median age at first concern of 2.0 years falls within this international range, yet 46.2% of families were in the “1–2 Years” delay category or longer. Earlier national evidence points in the same direction. A clinical review of 84 children reported a mean age at diagnosis of 3.8 years despite nearly all parents having noticed developmental delay before 30 months [17]. Regional evidence from Saudi Arabia reported a median age at diagnosis of 3.0 years [27]. Together, these findings place the Jordanian experience within a broader regional and international pattern while highlighting the substantial proportion of families experiencing prolonged delay.

Early signs initially not acted upon because they were interpreted as part of normal development were reported by 47.7% of caregivers and were the first pathway variable to produce a significant improvement in model fit. The association remained evident in the fully adjusted model (AOR = 2.21), under multiple imputation (AOR = 1.96), and in 99.5% of bootstrap resamples. This finding is consistent with qualitative evidence from the United Kingdom describing a period in which parents experienced developmental concerns without initially raising them with professionals, often because they could not clearly articulate what was concerning them [6]. Comparable accounts describe a phase of initial watchful waiting during which caregivers monitor developmental differences before seeking evaluation [28]. Cross-cultural research also indicates that judgments about when developmental differences warrant professional attention are shaped partly by social and cultural context [29]. The Jordanian context has similarly been described as involving parental tolerance of developmental variability and attribution of early differences to characteristics such as thoughtfulness or timidity [30]. In the present study, reporting that early signs were initially not acted upon because they were interpreted as part of normal development was associated with a longer diagnostic pathway independently of maternal education and household income. This finding should not be interpreted as attributing responsibility for delay to caregivers. The study did not assess whether families had received developmental guidance or reassurance from others, had access to routine developmental surveillance, or had realistic access to appropriate services when concerns first emerged. The retrospective design also precludes causal inference.

Previous professional reassurance that the individual did not have autism was reported by 38.0% of caregivers and was the most consistent correlate of longer diagnostic delay across the robustness analyses. The association was present in the primary ordinal model, in all three binary outcome groupings, under multiple imputation, and in the partial proportional-odds analysis. It was also retained in analyses using alternative symptom and missing-data specifications, and its estimated direction was positive in 100% of bootstrap resamples. This consistency identifies previous professional reassurance as an important provider-related pathway marker, although the retrospective design does not establish that reassurance itself caused subsequent delay. This finding is consistent with previous evidence showing that reassuring or passive professional responses to parental concern are associated with longer diagnostic pathways than proactive responses [10]. Qualitative studies provide a plausible explanation: parents who reported premature reassurance have described diminished confidence in their own observations and difficulties in subsequent interactions with healthcare professionals [6]. When developmental concerns persist, reassurance may therefore be most appropriately accompanied by explicit safety-netting, planned reassessment, and referral when indicated rather than being treated as diagnostic resolution. The study did not identify the specialty of the professional who provided reassurance. Therefore, the finding is relevant to provider-facing training across primary care and other first contact settings rather than being attributable specifically to primary care.

The exploratory analysis of caregiver-attributed barriers supported a related interpretation. Of 11 barriers examined, previous misdiagnosis was the only factor that remained statistically significant after Benjamini–Hochberg correction (adjusted OR = 2.21, 95% CI: 1.35–3.61, adjusted *p* = 0.017). Previous misdiagnosis has also been associated with prolonged diagnostic delay in Chinese cohorts [22,24], and a multinational review reported that a substantial proportion of autistic individuals receive at least one incorrect diagnosis before autism is identified [31]. In the present cohort, 71.6% of caregivers reported a prior specialist evaluation and 28.6% reported more than three evaluations before diagnosis. Related evidence also indicates that families may consult several professionals before a diagnostic conclusion is reached [32]. Taken together, these findings suggest that prolonged diagnostic pathways may reflect not only difficulties reaching professional care but also repeated professional contact that does not result in diagnostic resolution.

Definite difficulty obtaining appointments was reported by 31.2% of caregivers. Within the appointment difficulty variable, the Yes-versus-No contrast was associated with higher odds of longer diagnostic delay (AOR = 1.81, 95% CI: 1.08–3.02). A similar estimate was observed under multiple imputation (AOR = 2.10, 95% CI: 1.28–3.45). However, the appointment difficulty variable as a whole did not reach the conventional threshold for statistical significance in the global test (*p* = 0.070), so this coefficient-level finding should be interpreted cautiously. The pattern may be concentrated among caregivers reporting definite difficulty rather than reflecting a simple graded relationship. Such a finding is plausible within the structure of Jordanian services, which span the Ministry of Health, Royal Medical Services, university hospitals, the private sector, and refugee-serving agencies [33]. Diagnostic capacity remains concentrated within a limited number of specialist services. Previous Jordanian research has also described service fragmentation, financial pressures, and variability in service availability and quality [19,34].

Several associations anticipated from prior research were not observed. Maternal education, household income, urban residence, age, and sex of the individual with ASD showed no independent association with diagnostic delay. This differs from studies reporting socioeconomic gradients in diagnostic timing. In Latin American and Caribbean data, public health coverage was an important determinant [23], while Chinese evidence linked hospital distance and related enabling resources with diagnostic timeliness [24]. It is, however, consistent with a Saudi study that likewise found no significant relationship between family sociodemographic characteristics and age at first diagnosis [27]. One possible explanation is restricted socioeconomic variation within the present sample: 83.3% of families reported monthly household income of 600 Jordanian dinars or less, limiting the ability to detect income gradients. A second possibility is that common system-level constraints may reduce observable differences between socioeconomic groups when access to diagnostic capacity is restricted across much of the population. These explanations remain interpretive and require confirmation in samples with broader socioeconomic representation.

The absence of an independent sex effect also differs from the literature reporting later diagnosis among autistic girls [35]. The estimate in the present study was directionally opposite but did not reach statistical significance (AOR = 1.60, 95% CI: 0.94–2.73). Males constituted 78.1% of the sample, limiting statistical power for sex-specific inference. Evidence suggests that later diagnosis among autistic girls may reflect differences in clinical presentation, compensatory or camouflaging behaviors, and referral expectations. It may also reflect the historical development of diagnostic instruments based predominantly on male samples. The literature is not entirely consistent, however, with some cohorts reporting no association between sex and earlier diagnosis. Given the limited number of girls in the present sample, these mechanisms could not be examined in detail. Future studies with greater female representation and more detailed characterization of clinical presentation and referral pathways are needed to clarify sex-related differences in diagnostic timing.

Neither age at first concern nor the symptom-domain count was independently associated with delay, and the recognition block as a whole did not improve model fit over background characteristics alone (*p* = 0.562). This finding appears to differ from studies identifying child-level clinical characteristics as important determinants of age at diagnosis [22,23,27]. However, the difference in outcome definition is important. Studies modeling age at diagnosis capture both when developmental differences first become apparent and what happens afterward. By contrast, the present study examined the interval beginning after developmental concerns were already recognized. The findings therefore suggest that, within this cohort, the timing and breadth of caregiver-reported recognition were not strongly associated with how long diagnostic confirmation subsequently required. Caregiver response and healthcare-system factors appeared more informative for the post-recognition interval. Because predictor-specific diagnostics indicated variation across cumulative response-category groupings for age at first concern (*p* = 0.011), however, this result should be interpreted cautiously.

An important finding of this analysis was the association between the route of first professional contact and subsequent diagnostic delay. Compared with pediatrician first contact, entry through a speech or learning center was associated with longer delay (AOR = 2.27), as was entry through other services (AOR = 2.72). First contact with a general practitioner or autism center was not significantly associated with longer delay. This finding is consistent with evidence suggesting that the type of service through which developmental concerns first enter the healthcare or educational system may influence subsequent diagnostic progression. In a Chinese cohort, children with typical language competence experienced longer diagnostic delay, which the authors interpreted as reflecting greater attention to language milestones than to social-communication differences [24]. The same study found that regular well-child visits shortened delay, suggesting that entry through services incorporating developmental surveillance may be consequential [24]. Speech delay was reported by 83.3% of caregivers in the present cohort and was also the most frequent reason for presentation in an earlier Jordanian series [17], making speech-focused services a plausible early destination for many families. Grouping-specific analyses further showed that first contact through a speech or learning center had its strongest association for the “1–2 Years or longer” grouping (AOR = 3.23, 95% CI: 1.52–6.89). However, this association should be interpreted cautiously because residual confounding remains possible. In the individual-symptom sensitivity analysis, first contact through a speech or learning center remained associated with longer diagnostic delay after direct adjustment for speech delay and the other reported symptom domains (AOR = 2.20, 95% CI: 1.17–4.16, *p* = 0.015). This reduces concern that the association was explained solely by the measured symptom profile. However, residual confounding remains possible because clinical severity, functional language level, cognitive ability, and other clinical characteristics were not measured. Therefore, the observed association does not establish that first contact through a speech or learning center itself contributed to longer diagnostic delay.

A further methodological contribution of the study is the use of sequential nested models organized according to the temporal stages of the diagnostic pathway. Analyses of diagnostic timing frequently include family-level and system-level variables within a single multivariable model. Although such models provide valid adjusted associations, they provide less information about the incremental contribution of conceptually grouped pathway variables to overall model fit. Fitting five nested models on a common analytic sample allowed changes in model fit to be attributed to the addition of successive pathway blocks rather than to changes in the participants included. In this analysis, background and recognition contributed little additional explanatory value, while caregiver response, help-seeking route, and access/professional response produced sequential improvements in model fit. This approach may therefore be useful in other settings where diagnostic delay is measured in ordered categories and exact event dates are unavailable. The order of variable entry was based on the proposed analytical framework. Therefore, the models do not establish when these effects occurred during an individual diagnostic journey.

The findings suggest several potential areas for intervention across the diagnostic pathway. Caregiver-facing strategies may complement, rather than substitute for, provider- and system-level improvements by supporting recognition of social-communication features in addition to language delay and encouraging reassessment when developmental concerns persist. Provider-facing strategies could include training in structured referral criteria and standardized developmental and autism-specific screening. They could also include explicit safety-netting when reassurance is given, with planned reassessment or referral if developmental concerns persist. Standardized autism-specific screening at 18 and 24 months within routine child-health contacts provides an established framework [11], while regional evidence supports the feasibility of integrating Arabic-language screening tools into primary care [36,37]. Importantly, the findings concerning previous professional reassurance, repeated specialist contact, and access-related difficulties indicate that delayed diagnosis should not be understood primarily as a consequence of insufficient caregiver awareness. Expanding diagnostic capacity alone may also be insufficient unless referral pathways support timely movement toward diagnostic resolution. Scalable and culturally appropriate diagnostic approaches outside a limited number of tertiary centers may therefore warrant consideration.

## 5. Strengths

This study has several methodological strengths. The outcome was defined as the recognition-to-diagnosis interval rather than age at diagnosis, allowing the analysis to focus specifically on the period after developmental concerns had already emerged. The sequential nested design fitted five models on a single common complete-case sample of 299 participants, ensuring that changes in fit reflected the addition of pathway blocks rather than changes in the analytic sample. Model progression was assessed using likelihood-ratio tests, Akaike and Bayesian information criteria, and two pseudo-R^2^ measures. For multi-category predictors, block-level likelihood-ratio tests were used rather than relying solely on individual coefficients. Ordinal logistic regression was appropriate for an outcome recorded in ordered categories of unequal width rather than as exact continuous event times.

The robustness analyses were also extensive. Binary models were fitted for three clinically interpretable outcome groupings to assess whether associations differed across delay categories. Multiple imputation across 20 datasets extended the analysis to all 338 participants with known diagnostic timing and reproduced the five highlighted coefficient-level associations. Bootstrap resampling across 200 replications demonstrated directional stability in 98.0% to 100% of resamples for the highlighted findings. The proportional-odds assumption was formally evaluated, and a modest departure was identified (χ^2^ = 69.61, df = 51, *p* = 0.043). This was examined further using predictor-specific diagnostics, grouping-specific models, and a partial proportional-odds generalized ordered logistic sensitivity analysis. Participants with unknown diagnostic timing were also compared systematically with those with known timing. Finally, recruitment through autism centers, special-education settings, clinical services, and community networks included urban, rural, and Bedouin communities across multiple governorates. This provided broader geographic coverage than the single-center clinical series that has characterized much of the previous Jordanian literature [17].

## 6. Limitations

Several limitations should be considered when interpreting these findings. The study was cross-sectional, and all pathway variables were reported retrospectively. Consequently, the observed associations should be interpreted as markers of the diagnostic pathway rather than causal effects. Recall and reconstruction are possible. Caregivers who experienced prolonged delay may remember early concerns or professional responses differently from those whose children were diagnosed promptly. Recall bias is a recognized limitation of comparable studies examining first concerns and diagnostic timing [22,25,26]. The mean age of individuals with ASD in this sample was 10.0 years, so some caregivers were recalling events from several years earlier. In addition, 12.0% of the cohort could not recall the diagnostic interval. Diagnostic delay was recorded in ordered categories rather than exact months, limiting temporal precision and precluding survival-analysis approaches used in studies with exact event dates [22]. Diagnostic status was not independently re-verified against medical documentation or through a study-administered standardized instrument. The diagnosing specialty or credential was also not uniformly recorded across sites. Some diagnostic heterogeneity across sites and specialists therefore cannot be excluded. No age restriction was applied, and year of diagnosis was not recorded. The cohort may therefore span periods with different levels of autism awareness, referral practices, and diagnostic capacity in Jordan. These historical changes may have contributed to variation in caregiver-reported diagnostic delay.

Sampling and measurement also impose limitations. Nonprobability convenience sampling was used, and part of the sample was recruited through an open electronic link. Therefore, a conventional response rate could not be calculated, and the sample may have been more likely to include families already engaged with diagnostic or educational services. Of the 389 submitted responses, 73.0% were completed online, and potential mode effects between online and face-to-face administration cannot be excluded. Recruitment through service-linked and community networks may also limit generalization to ASD families not already connected to such services. Individuals who had not yet received an ASD diagnosis were necessarily absent, so the study does not capture the most extreme unresolved diagnostic pathways. Two distinct sources of missingness should be considered. First, the primary models included 299 complete cases among 338 participants with known diagnostic timing, raising the possibility of selection bias related to covariate values unavailable under the primary complete-case coding. However, multiple imputation extended the analysis to all 338 participants with known timing and produced similar estimates. It also reproduced the five highlighted coefficient-level associations, reducing concern that the complete-case restriction materially drove the findings. Second, multiple imputation did not address the 46 participants with unknown diagnostic timing because the outcome itself was not imputed. Participants with known and unknown timing differed in maternal education (*p* = 0.007), caregiver response (*p* = 0.004), appointment difficulty (*p* < 0.001), and professional reassurance (*p* = 0.003), indicating that outcome missingness may have been selective. Therefore, selection related to unavailable diagnostic timing cannot be excluded. The present study did not measure ASD clinical severity, functional language or nonverbal status, cognitive ability, co-occurring intellectual disability, or other co-occurring conditions. These unmeasured clinical characteristics could influence both caregiver response to early developmental concerns and the type of service contacted first, potentially causing residual confounding. The symptom-domain list was a pragmatic, non-validated caregiver-report measure limited to six developmental concerns and was not intended to be exhaustive. Other potentially relevant concerns, such as sensory sensitivities, developmental regression, and motor abnormalities, were not assessed. Individual reported symptom domains were examined in sensitivity analyses. Although the reported symptom domains captured the presence of specific developmental concerns, they did not provide standardized measures of symptom severity, functional language, or intellectual functioning. Residual confounding should therefore be considered when interpreting associations with caregiver response and first contact route. Individuals with more pronounced developmental or communication impairments may follow different help-seeking and referral pathways than those with subtler or predominantly language-focused presentations. In addition, 83.3% of the sample reported a household income of 600 Jordanian dinars or less, limiting inference regarding socioeconomic gradients. The predominance of males also limits sex-specific analysis. These limitations should be addressed in prospective studies that incorporate exact event timing, standardized measures of ASD severity, functional language, and intellectual functioning, co-occurring conditions, and broader population-based recruitment.

## 7. Synthesis

Taken together, the findings indicate that, within the post-recognition interval examined in this study, caregiver-reported help-seeking experiences and healthcare-pathway variables were more strongly associated with diagnostic delay than the background and recognition variables included in the models. Caregivers identified concerns at a median age of 2.0 years, yet nearly half of those with known timing were in the “1–2 Years” delay category or longer. Background characteristics and recognition variables explained little of this variation, whereas caregiver response, help-seeking route, and access/professional response progressively improved model fit.

Three potentially modifiable pathway features emerged. These were caregivers initially interpreting early developmental signs as normal variation, entry through services that may address presenting symptoms without immediately prompting broader developmental assessment, and professional reassurance that is not followed by diagnostic resolution. These findings identify plausible targets for referral-system improvement and provider-facing interventions, complemented by caregiver-facing information and support, but they should not be interpreted as demonstrating causal effects. Prospective studies incorporating exact event timing, clinical severity and cognitive characterization, and individuals not yet diagnosed will be necessary to determine whether modifying these pathway features shortens the recognition-to-diagnosis interval in practice.

## 8. Conclusions

This study indicates that, in Jordan, the length of the recognition-to-diagnosis interval for ASD was associated with factors that operate after developmental concerns are first recognized, rather than with the timing or breadth of recognition itself. Caregivers identified concerns at a median age of 2.0 years, yet nearly half of those with known diagnostic timing were in the “1–2 Years” delay category or longer. Background and recognition variables contributed relatively little to model performance, whereas the addition of caregiver response, first contact route, and access/professional response factors was associated with incremental improvements in model fit. Nevertheless, the overall explanatory capacity of the final model remained modest, indicating that substantial variability in diagnostic delay was not captured by the measured variables.

Longer diagnostic delay was independently associated with three pathway factors. These were caregivers reporting that early signs had initially not been acted upon because they were interpreted as part of normal development, first contact through a speech or learning center or other service rather than a pediatrician, and caregiver-reported previous professional reassurance that the individual did not have ASD. Definite appointment difficulty also showed a significant Yes-versus-No contrast. However, the appointment difficulty variable as a whole was not statistically significant in the global test (*p* = 0.070) and should therefore be interpreted cautiously. Caregiver-reported previous professional reassurance was the most consistent correlate across sensitivity analyses, while previous misdiagnosis was the only exploratory caregiver-attributed barrier to remain significant after multiplicity correction.

These findings support provider- and system-level improvements, including provider training, clearer referral pathways, explicit safety-netting and follow-up when reassurance is provided, and expansion of accessible diagnostic capacity, complemented by caregiver-facing information and support. Because the study was cross-sectional and retrospective, these associations should be interpreted as pathway markers rather than causal effects. Prospective studies with exact event timing and more detailed clinical characterization are needed to determine whether interventions targeting these pathway features can shorten the recognition-to-diagnosis interval.

## Figures and Tables

**Table 1:** Sociodemographic and clinical characteristics of the study cohort (N = 384).

Characteristic	Value
**Characteristics of individuals with ASD**	
Age, mean ± SD, years	10.0 ± 5.0
Age, median (IQR), years	9 (6–13)
Male sex	300 (78.1%)
Female sex	84 (21.9%)
**School setting**	
Special-needs education center	187 (48.7%)
Does not attend school	99 (25.8%)
Public school	55 (14.3%)
Private school	43 (11.2%)
**Maternal education**	
Elementary	21 (5.5%)
Middle school	48 (12.7%)
High school	136 (35.9%)
Diploma	57 (15.0%)
Bachelor’s degree	99 (26.1%)
Postgraduate	18 (4.7%)
**Monthly household income**	
<300 JOD	158 (41.1%)
300–600 JOD	162 (42.2%)
601–1000 JOD	48 (12.5%)
>1000 JOD	16 (4.2%)
**Residence**	
City	269 (70.1%)
Rural	99 (25.8%)
Bedouin/desert	16 (4.2%)
**Health insurance**	
Yes	316 (82.3%)
No	68 (17.7%)
**Family history of ASD**	
Yes	27 (7.0%)
No	337 (87.8%)
Maybe	20 (5.2%)

Footnotes: data are presented as n (%) unless otherwise specified. Maternal education was available for 379 participants; all other characteristics in this table were available for the full cohort. ASD = autism spectrum disorder; JOD = Jordanian dinar; SD = standard deviation; IQR = interquartile range.

**Table 2:** Recognition-to-diagnosis pathway and diagnostic delay.

**Panel A.** Recognition-to-Diagnosis Pathway Characteristics (N = 384).	
**Pathway Characteristic**	**Value**
**Recognition**	
Age at first concern, median (IQR), years	2.0 (1.5–3.0)
Speech delay	320 (83.3%)
Eye contact problems	254 (66.1%)
Social interaction problems	255 (66.4%)
Repetitive behaviors	220 (57.3%)
Behavioral problems	200 (52.1%)
Cognitive/developmental delay	146 (38.0%)
**Caregiver response to early signs**	
Early signs initially not acted upon: Yes	183 (47.7%)
Early signs initially not acted upon: Maybe	53 (13.8%)
Early signs initially not acted upon: No	148 (38.5%)
**First help-seeking route**	
Pediatrician	136 (35.5%)
General practitioner	59 (15.4%)
Autism center	87 (22.7%)
Speech/learning center	68 (17.8%)
Other service	33 (8.6%)
**Evaluation before diagnosis**	
Prior specialist evaluation: Yes	275 (71.6%)
Prior specialist evaluation: No	90 (23.4%)
Prior specialist evaluation: Unknown	19 (4.9%)
Number of evaluations: Once	65 (16.9%)
Number of evaluations: Twice	62 (16.1%)
Number of evaluations: Three times	45 (11.7%)
Number of evaluations: >3 times	110 (28.6%)
Number of evaluations: Unknown	102 (26.6%)
**Professional response and access**	
Previous professional reassurance: Yes	146 (38.0%)
Previous professional reassurance: No	214 (55.7%)
Previous professional reassurance: Unknown	24 (6.2%)
Appointment difficulty: No	147 (38.3%)
Appointment difficulty: Sometimes	99 (25.8%)
Appointment difficulty: Yes	120 (31.2%)
Appointment difficulty: Unknown	18 (4.7%)
**Panel B.** Diagnostic Delay Distribution and Cumulative Response-Category Groupings.	
**Delay category or grouping**	**n/N (%)**
**Mutually exclusive delay categories among participants with known timing (N = 338)**	
Less than 3 Months	87/338 (25.7%)
3–6 Months	46/338 (13.6%)
6 Months to 1 Year	49/338 (14.5%)
1–2 Years	75/338 (22.2%)
More than 2 Years	81/338 (24.0%)
**Cumulative response-category groupings among participants with known timing (N = 338)**	
6 Months to 1 Year or longer	205/338 (60.7%)
1–2 Years or longer	156/338 (46.2%)
>2 years	81/338 (24.0%)
**Outcome availability in the full cohort (N = 384)**	
“Do not remember” diagnostic interval	46/384 (12.0%)

Footnotes: data are presented as n (%) unless otherwise specified. Age at first developmental concern was available for 381 participants. The first contact route was available to 383 participants; percentages for first contact categories are based on those 383 responses. The “other service” category (n = 33) included neurologist/pediatric neurologist contacts (n = 8), nonspecified physicians (n = 6), diagnostic/disability or intelligence-assessment centers (n = 7), hospital/emergency or government-facility contacts (n = 4), mixed or multiple-service responses (n = 5), and three additional individual responses (hearing assessment before ASD screening, speech therapist, and a non-provider response referring to seizures). Other percentages in Panel A are based on N = 384. ASD = autism spectrum disorder; IQR = interquartile range. Prior specialist evaluation and number of evaluations before diagnosis were assessed as separate items; the latter was not restricted to specialist evaluations. The five mutually exclusive delay categories sum to 100% among the 338 participants with known timing. Cumulative groups overlap: participants in the 1–2 Years or longer group are also included in the 6 Months to 1 Year or longer group, and participants in the >2-year group are included in both cumulative groups.

**Table 3:** Sequential nested models of longer diagnostic delay.

**Panel A.** Model Progression.						
**Model**	**Block Added**	**AIC**	**BIC**	**McFadden R^2^**	**Nagelkerke R^2^**	**LR *p* Vs. Previous**
M1	Background characteristics	953.1	**986.4**	0.006	0.021	-
M2	Recognition	956.0	996.7	0.008	0.025	0.562
M3	Caregiver response	946.7	994.8	0.022	0.069	**0.001**
M4	Help-seeking route	941.2	1007.8	0.038	0.118	**0.008**
M5	Access and professional response	**927.3**	1005.0	0.059	0.178	**<0.001**
**Panel B.** Fully Adjusted Ordinal Logistic Regression Model (M5; N = 299).						
**Predictor**				**AOR (95% CI)**		***p***
**Background characteristics**						
Age of individual with ASD, per year				1.04 (0.99–1.09)		0.124
Male sex				1.60 (0.94–2.73)		0.083
Maternal education, per category				0.94 (0.77–1.14)		0.513
Household income, per category				1.14 (0.85–1.54)		0.385
Urban residence				0.75 (0.46–1.20)		0.228
**Recognition**						
Age at first concern, per year				0.96 (0.78–1.18)		0.688
Symptom-domain count, per domain				0.94 (0.83–1.06)		0.303
**Caregiver response**						
Early signs initially not acted upon: Maybe vs. No				1.31 (0.61–2.78)		0.488
Early signs initially not acted upon: Yes vs. No				**2.21 (1.38–3.54)**		**0.001**
**Help-seeking route**						
GP vs. pediatrician				1.27 (0.67–2.40)		0.464
Autism center vs. pediatrician				1.02 (0.59–1.78)		0.939
Speech/learning center vs. pediatrician				**2.27 (1.20–4.27)**		**0.011**
Other service vs. pediatrician				**2.72 (1.22–6.09)**		**0.015**
Prior specialist evaluation				0.76 (0.47–1.25)		0.288
**Access and professional response**						
Appointment difficulty: Sometimes vs. No				1.41 (0.82–2.40)		0.212
Appointment difficulty: Yes vs. No				**1.81 (1.08–3.02)**		**0.023**
Previous professional reassurance				**2.18 (1.40–3.38)**		**<0.001**

Footnotes: all models used the same complete-case sample (N = 299). M1 included age and sex of the individual with ASD, maternal education, household income, and urban residence; M2 added age at first concern and symptom-domain count; M3 added caregiver response; M4 added first contact route and prior specialist evaluation; M5 added appointment difficulty and previous professional reassurance. Lower AIC and BIC values indicate better parsimony-adjusted fit. LR *p* compares each model with the preceding model. Bold indicates the lowest AIC/BIC value and LR tests with *p* < 0.05. AOR = adjusted odds ratio; CI = confidence interval. Odds ratios greater than 1 indicate higher odds of belonging to a longer diagnostic delay category. Pediatrician and No were reference categories as applicable; female sex and non-urban residence were reference categories for binary predictors. Maternal education and household income were modeled per increasing category. Bold indicates *p* < 0.05. Because these data were cross-sectional and retrospectively reported, adjusted associations represent diagnostic-pathway markers and should not be interpreted as causal effects. Given the modest departure from the proportional-odds assumption for the overall model, these ordinal AORs represent an average association across the ordered delay categories rather than a uniform effect across all cumulative response-category groupings; see Table 4 for grouping-specific estimates.

**Table 4:** Robustness of key adjusted associations across alternative delay definitions and multiple imputation.

Predictor	Ordinal (CC; N = 299)	6 Months to 1 Year or Longer	1–2 Years or Longer	>2 Years	Ordinal (MI; N = 338)
**Caregiver response**					
Early signs initially not acted upon: Maybe vs. No	1.31 (0.61–2.78)	1.36 (0.59–3.12)	1.35 (0.57–3.21)	**3.58 (1.30–9.85)**	1.19 (0.59–2.39)
Early signs initially not acted upon: Yes vs. No	**2.21 (1.38–3.54)**	**2.29 (1.31–4.00)**	1.69 (0.96–2.96)	**2.54 (1.25–5.18)**	**1.96 (1.26–3.05)**
**Help-seeking route**					
GP vs. pediatrician	1.27 (0.67–2.40)	1.45 (0.67–3.14)	1.21 (0.57–2.59)	1.76 (0.74–4.17)	1.39 (0.76–2.54)
Autism center vs. pediatrician	1.02 (0.59–1.78)	1.00 (0.52–1.94)	1.21 (0.62–2.38)	0.90 (0.38–2.17)	1.06 (0.63–1.79)
Speech/learning vs. pediatrician	**2.27 (1.20–4.27)**	1.95 (0.89–4.24)	**3.23 (1.52–6.89)**	1.66 (0.71–3.87)	**2.08 (1.14–3.79)**
Other route vs. pediatrician	**2.72 (1.22–6.09)**	2.22 (0.80–6.20)	**3.74 (1.41–9.92)**	2.26 (0.79–6.48)	**3.00 (1.42–6.34)**
Prior specialist evaluation	0.76 (0.47–1.25)	0.67 (0.36–1.24)	0.82 (0.45–1.50)	0.77 (0.37–1.60)	0.79 (0.48–1.28)
**Access and professional response**					
Appointment difficulty: Sometimes vs. No	1.41 (0.82–2.40)	1.34 (0.70–2.55)	1.08 (0.56–2.08)	2.07 (0.94–4.56)	1.65 (0.99–2.73)
Appointment difficulty: Yes vs. No	**1.81 (1.08–3.02)**	1.86 (1.00–3.46)	**2.04 (1.11–3.75)**	**2.33 (1.10–4.94)**	**2.10 (1.28–3.45)**
Previous professional reassurance	**2.18 (1.40–3.38)**	**2.80 (1.62–4.83)**	**2.10 (1.24–3.56)**	**1.89 (1.01–3.52)**	**1.92 (1.26–2.94)**

Footnotes: cells show AOR (95% CI). The complete-case (CC) ordinal and binary models used N = 299. The ordinal multiple imputation (MI) model included all 338 participants with known diagnostic timing across 20 imputed datasets; multiple imputation addressed missing covariates, while the diagnostic delay outcome itself was not imputed. The “6 Months to 1 Year or longer” and “1–2 Years or longer” columns represent cumulative response-category groupings; >2 years was an exploratory extreme-delay analysis. Pediatrician and No were reference categories as applicable. Bold indicates *p* < 0.05. The appointment difficulty Yes-versus-No estimate for the “6 Months to 1 Year or longer” outcome had *p* = 0.0500 and is therefore not bolded under the prespecified *p* < 0.05 criterion. AOR = adjusted odds ratio; CI = confidence interval; CC = complete case; MI = multiple imputation.

## Data Availability

The data presented in this study are available on request from the corresponding author. The data are not publicly available due to privacy and ethical reasons.

## Supplementary Materials

The following supporting information can be downloaded at: https://www.mdpi.com/article/10.3390/children13091228/s1, Table S1: Caregiver-Attributed Barriers and Longer Diagnostic Delay; Table S2: Comparison of Participants With Known Versus Unknown Diagnostic Timing; Table S3: Complete Coefficient Estimates Across Sequential Models M1–M5; Table S4: Partial Proportional-Odds Generalized Ordered Logistic Regression Sensitivity Analysis (N = 299); Table S5: Sensitivity Analysis Replacing Symptom-Domain Count With Individual Reported Symptom Domains (N = 299); Table S6: Sensitivity Analyses Accounting for Explicit Unknown/Do Not Remember Responses.

## References

[1] Zeidan J., Fombonne E., Scorah J., Ibrahim A., Durkin M.S., Saxena S., Yusuf A., Shih A., Elsabbagh M. (2022). Global prevalence of autism: A systematic review update. Autism Res..

[2] World Health Organization (2025). Autism. https://www.who.int/news-room/fact-sheets/detail/autism-spectrum-disorders.

[3] Zwaigenbaum L., Bauman M.L., Choueiri R., Kasari C., Carter A., Granpeesheh D., Mailloux Z., Roley S.S., Wagner S., Fein D. (2015). Early Intervention for Children with Autism Spectrum Disorder Under 3 Years of Age: Recommendations for Practice and Research. Pediatrics.

[4] Daniels A.M., Mandell D.S. (2014). Explaining differences in age at autism spectrum disorder diagnosis: A critical review. Autism.

[5] Van’t Hof M., Tisseur C., van Berckelear-Onnes I., van Nieuwenhuyzen A., Daniels A.M., Deen M., Hoek H.W., Ester W.A. (2021). Age at autism spectrum disorder diagnosis: A systematic review and meta-analysis from 2012 to 2019. Autism.

[6] Ryan S., Salisbury H. (2012). ‘You know what boys are like’: Pre-diagnosis experiences of parents of children with autism spectrum conditions. Br. J. Gen. Pract..

[7] Khalil A., Yatcilla J., Christie N., Zhou X. (2025). A Systematic Review of Help-Seeking Barriers for Racial-Ethnic Minority Caregivers Accessing Autism Diagnostic and Intervention Services. J. Racial Ethn. Health Disparities.

[8] Weitlauf A.S., Miceli A., Vehorn A., Dada Y., Pinnock T., Harris J.W., Hine J., Warren Z. (2024). Screening, Diagnosis, and Intervention for Autism: Experiences of Black and Multiracial Families Seeking Care. J. Autism Dev. Disord..

[9] Gordon-Lipkin E., Foster J., Peacock G. (2016). Whittling Down the Wait Time: Exploring Models to Minimize the Delay from Initial Concern to Diagnosis and Treatment of Autism Spectrum Disorder. Pediatr. Clin. N. Am..

[10] Zuckerman K.E., Lindly O.J., Sinche B.K. (2015). Parental Concerns, Provider Response, and Timeliness of Autism Spectrum Disorder Diagnosis. J. Pediatr..

[11] Hyman S.L., Levy S.E., Myers S.M., Kuo D.Z., Apkon S., Davidson L.F., Ellerbeck K.A., Foster J.E., Noritz G.H., Council on Children with Disabilities, Section on Developmental and Behavioral Pediatrics (2020). Identification, Evaluation, and Management of Children With Autism Spectrum Disorder. Pediatrics.

[12] Penner M., Anagnostou E., Ungar W.J. (2018). Practice patterns and determinants of wait time for autism spectrum disorder diagnosis in Canada. Mol. Autism.

[13] Cermak C.A., Rapley J., Fournier S., Penner M. (2026). Barriers and Innovations Towards Accessing an Autism Diagnosis in Rural Northern Ontario: A Qualitative Study. Child Care Health Dev..

[14] Estrin G.L., Milner V., Spain D., Happé F., Colvert E. (2021). Barriers to Autism Spectrum Disorder Diagnosis for Young Women and Girls: A Systematic Review. Rev. J. Autism Dev. Disord..

[15] Divan G., Bhavnani S., Leadbitter K., Ellis C., Dasgupta J., Abubakar A., Elsabbagh M., Hamdani S.U., Servili C., Patel V. (2021). Annual Research Review: Achieving universal health coverage for young children with autism spectrum disorder in low- and middle-income countries: A review of reviews. J. Child Psychol. Psychiatry.

[16] Matos M.B., Bara T.S., Cordeiro M.L. (2022). Autism Spectrum Disorder Diagnoses: A Comparison of Countries with Different Income Levels. Clin. Epidemiol..

[17] Masri A.T., Al Suluh N., Nasir R. (2013). Diagnostic delay of autism in Jordan: Review of 84 cases. Libyan J. Med..

[18] Hyassat M., Al-Makahleh A., Rahahleh Z., Al-Zyoud N. (2023). The Diagnostic Process for Children with Autism Spectrum Disorder: A Preliminary Study of Jordanian Parents’ Perspectives. Children.

[19] Masri A.T., Nasir A.K., Irshaid A.G., Irshaid F.Y., Alomari F.K., Khatib F.A., Al-Qudah A.A., Nafi O.A., Almomani M.A., Bashtawi M.A. (2023). Autism services in low-resource areas. Neurosciences.

[20] Santomauro D.F., Erskine H.E., Herrera A.M.M., Miller P.A., Shadid J., Hagins H., Addo I.Y., Adnani Q.E.S., Ahinkorah B.O., Ahmed A. (2025). The global epidemiology and health burden of the autism spectrum: Findings from the Global Burden of Disease Study 2021. Lancet Psychiatry.

[21] Shaw K.A., Williams S., Patrick M.E., Valencia-Prado M., Durkin M.S., Howerton E.M., Ladd-Acosta C.M., Pas E.T., Bakian A.V., Bartholomew P. (2025). Prevalence and Early Identification of Autism Spectrum Disorder Among Children Aged 4 and 8 Years — Autism and Developmental Disabilities Monitoring Network, 16 Sites, United States, 2022. Morb. Mortal. Wkly. Rep..

[22] Zhu F.-L., Ji Y., Wang L., Xu M., Zou X.-B. (2025). Current situation and influencing factors of Chinese children’s diagnosis delay in autism. J. Neurodev. Disord..

[23] Montiel-Nava C., Montenegro M.C., Ramirez A.C., Valdez D., Rosoli A., Garcia R., Garrido G., Cukier S., Rattazzi A., Paula C.S. (2024). Age of autism diagnosis in Latin American and Caribbean countries. Autism.

[24] Li Z., Niu X., Wong P.C.M., Zhang H., Wang L. (2025). Factors influencing timely diagnosis of autism in China: An application of Andersen’s behavioral model of health services use. BMC Psychiatry.

[25] Crane L., Chester J.W., Goddard L., Henry L.A., Hill E. (2016). Experiences of autism diagnosis: A survey of over 1000 parents in the United Kingdom. Autism.

[26] Gibbs V., Aldridge F., Sburlati E., Chandler F., Smith K., Cheng L. (2019). Missed opportunities: An investigation of pathways to autism diagnosis in Australia. Res. Autism Spectr. Disord..

[27] Alnemary F.M., Simon-Cereijido G., Aldhalaan H.M., Hernandez A., Alyahya A., Alenezi S. (2022). Factors associated with age of diagnosis of autism spectrum disorder among children in Saudi Arabia: New insights from a cross-sectional study. BMC Res. Notes.

[28] Smith-Young J., Chafe R., Audas R. (2020). “Managing the Wait”: Parents’ Experiences in Accessing Diagnostic and Treatment Services for Children and Adolescents Diagnosed With Autism Spectrum Disorder. Health Serv. Insights.

[29] Daley T.C. (2004). From symptom recognition to diagnosis: Children with autism in urban India. Soc. Sci. Med..

[30] Nasir A.K., Masri A.T., Shaheen S., Sayles H., Nasir L. (2026). Arabic Language Autism Diagnostic Interview (ALADIN): A Validation Study. J. Autism Dev. Disord..

[31] Fusar-Poli L., Brondino N., Politi P., Aguglia E. (2022). Missed diagnoses and misdiagnoses of adults with autism spectrum disorder. Eur. Arch. Psychiatry Clin. Neurosci..

[32] Goin-Kochel R.P., Mackintosh V.H., Myers B.J. (2006). How many doctors does it take to make an autism spectrum diagnosis?. Autism.

[33] Tamimi A., Al-Abbadi M., Tamimi I., Juweid M., Ahmad M., Tamimi F. (2024). The transformation of Jordan’s healthcare system in an area of conflict. BMC Health Serv. Res..

[34] AL Jabery M.A., Arabiat D.H., AL Khamra H.A., Betawi I.A., Jabbar S.K.A. (2014). Parental Perceptions of Services Provided for Children with Autism in Jordan. J. Child Fam. Stud..

[35] McDonnell C.G., DeLucia E.A., Hayden E.P., Penner M., Curcin K., Anagnostou E., Nicolson R., Kelley E., Georgiades S., Liu X. (2021). Sex Differences in Age of Diagnosis and First Concern among Children with Autism Spectrum Disorder. J. Clin. Child Adolesc. Psychol..

[36] Alawami A.H., Perrin E.C., Sakai C. (2019). Implementation of M-CHAT Screening for Autism in Primary Care in Saudi Arabia. Glob. Pediatr. Health.

[37] Mohamed F., Zaky E., Youssef A., Elhossiny R., Zahra S., Khalaf R., Youssef W., Wafiq A., Ibrahim R., Abd-Elhakim R. (2016). Screening of Egyptian toddlers for autism spectrum disorder using an Arabic validated version of M-CHAT; report of a community-based study (Stage I). Eur. Psychiatry.

