# Supplementary material for: From Recognition to Diagnosis: Caregiver Response, Help-Seeking Pathways, and Access-Related Factors Associated with Autism Diagnostic Delay in Jordan
Source: Children (Basel). 2026 Sep 11;13(9):1228. doi: 10.3390/children13091228 (PMC13605918; doi:10.3390/children13091228)
Supplement: Supplementary file 1 [file children-13-01228-s001.zip › children-4535547-supplementary.pdf]

Supplementary Material

Supplementary Table S1. Caregiver-Attributed Barriers and Longer Diagnostic Delay.

**Barrier**	**n (%)**	**Adjusted OR (95% CI)**	**BH-adjusted p**
Lack of knowledge/awareness	230 (59.9%)	1.50 (1.01-2.24)	.215
Lack of time / parents busy	67 (17.4%)	0.88 (0.53-1.46)	.730
Difficulty accessing healthcare	113 (29.4%)	1.50 (0.99-2.29)	.215
Delayed specialist appointments	78 (20.3%)	1.12 (0.71-1.77)	.730
Previous misdiagnosis	75 (19.5%)	**2.21 (1.35-3.61)**	**.017**
Financial cost	148 (38.5%)	1.14 (0.76-1.72)	.730
Fear/anxiety	110 (28.6%)	0.91 (0.60-1.39)	.730
Social stigma	58 (15.1%)	0.88 (0.52-1.47)	.730
Lack of community/family support	84 (21.9%)	1.31 (0.83-2.08)	.544
Cultural/religious reasons	22 (5.7%)	1.89 (0.75-4.79)	.493
Lack of trust in healthcare providers	46 (12.0%)	1.01 (0.56-1.81)	.971

Footnotes: Barrier frequencies, n (%), are based on the full eligible cohort (N = 384), whereas adjusted regression models used N = 334. Each barrier was examined in a separate adjusted ordinal logistic regression model including age and sex of the individual with ASD, maternal education, household income, and urban residence. Benjamini-Hochberg correction was applied across 11 barrier tests. Odds ratios greater than 1 indicate higher odds of longer diagnostic delay. Bold indicates statistical significance after multiplicity correction. BH = Benjamini-Hochberg; CI = confidence interval; OR = odds ratio.

Supplementary Table S2. Comparison of Participants With Known Versus Unknown Diagnostic Timing.

**Characteristic**	**Known timing**	**Unknown timing**	**p**
**Background characteristics**			
Age of individual with ASD, median (IQR), years	9.0 (6.0-13.0)	11.0 (8.0-13.75)	.161
Sex			.191
Male	268 (79.3%)	32 (69.6%)	
Female	70 (20.7%)	14 (30.4%)	
Maternal education			**.007**
Elementary	13 (3.9%)	8 (17.8%)	
Middle school	43 (12.9%)	5 (11.1%)	
High school	119 (35.6%)	17 (37.8%)	
Diploma	52 (15.6%)	5 (11.1%)	
Bachelor's	91 (27.2%)	8 (17.8%)	
Postgraduate	16 (4.8%)	2 (4.4%)	
Household income			.232
<300 JOD	135 (39.9%)	23 (50.0%)	
300-600 JOD	142 (42.0%)	20 (43.5%)	
601-1000 JOD	46 (13.6%)	2 (4.3%)	
>1000 JOD	15 (4.4%)	1 (2.2%)	
Residence			.079
City	242 (71.6%)	27 (58.7%)	
Rural	81 (24.0%)	18 (39.1%)	
Bedouin/desert	15 (4.4%)	1 (2.2%)	
**Caregiver response and pathway characteristics**			
Early signs initially not acted upon			**.004**

Characteristic	Known timing	Unknown timing	p
No	129 (38.2%)	19 (41.3%)	
Maybe	40 (11.8%)	13 (28.3%)	
Yes	169 (50.0%)	14 (30.4%)	
First professional/service			.095
Pediatrician	125 (37.1%)	11 (23.9%)	
General practitioner	53 (15.7%)	6 (13.0%)	
Autism center	73 (21.7%)	14 (30.4%)	
Speech/learning center	55 (16.3%)	13 (28.3%)	
Other	31 (9.2%)	2 (4.3%)	
Appointment difficulty			**<.001**
No	129 (38.2%)	18 (39.1%)	
Sometimes	91 (26.9%)	8 (17.4%)	
Yes	108 (32.0%)	12 (26.1%)	
Unknown	10 (3.0%)	8 (17.4%)	
Previous professional reassurance			**.003**
No	194 (57.4%)	20 (43.5%)	
Yes	128 (37.9%)	18 (39.1%)	
Unknown	16 (4.7%)	8 (17.4%)	

Footnotes: Known diagnostic timing was available for 338 participants and unknown timing for 46. Maternal education was available for 334 participants with known timing and 45 with unknown timing. First-contact route was available for 337 participants with known timing and 46 with unknown timing. Percentages are column percentages within timing-status groups and may therefore be based on available data for individual variables. Age was compared using the Mann-Whitney U test; categorical variables were compared using Pearson's chi-square or Fisher's exact test, as appropriate. Bold p values indicate p < .05. IQR = interquartile range; JOD = Jordanian dinar.

Supplementary Table S3. Complete Coefficient Estimates Across Sequential Models M1-M5.

Predictor	M1	M2	M3	M4	M5
Age of individual with ASD, per year	1.03 (0.99-1.08); .169	1.03 (0.98-1.07); .268	1.03 (0.99-1.08); .148	1.03 (0.99-1.08); .163	1.04 (0.99-1.09); .124
Male sex	1.42 (0.85-2.38); .179	1.46 (0.87-2.44); .154	1.53 (0.91-2.58); .106	1.52 (0.90-2.57); .116	1.60 (0.94-2.73); .083
Maternal education, per category	0.93 (0.77-1.12); .454	0.94 (0.77-1.13); .500	0.91 (0.75-1.11); .360	0.94 (0.77-1.14); .503	0.94 (0.77-1.14); .513
Household income, per category	1.06 (0.79-1.41); .699	1.05 (0.78-1.40); .757	1.08 (0.81-1.44); .590	1.09 (0.81-1.46); .559	1.14 (0.85-1.54); .385
Urban residence	0.74 (0.47-1.16); .186	0.76 (0.48-1.21); .250	0.78 (0.49-1.24); .298	0.75 (0.47-1.20); .226	0.75 (0.46-1.20); .228
Age at first concern, per year	-	1.06 (0.87-1.28); .584	0.97 (0.79-1.19); .773	0.97 (0.79-1.18); .734	0.96 (0.78-1.18); .688
Symptom-domain count, per domain	-	0.94 (0.83-1.06); .340	0.96 (0.85-1.08); .510	0.93 (0.82-1.05); .246	0.94 (0.83-1.06); .303
Early signs initially not acted upon: Maybe vs No	-	-	1.47 (0.71-3.06); .298	1.40 (0.66-2.97); .375	1.31 (0.61-2.78); .488
Early signs initially not acted upon: Yes vs No	-	-	**2.31 (1.46-3.64); <.001**	**2.53 (1.59-4.01); <.001**	**2.21 (1.38-3.54); .001**
First contact: GP vs pediatrician	-	-	-	1.18 (0.63-2.23); .604	1.27 (0.67-2.40); .464
First contact: autism center vs pediatrician	-	-	-	0.93 (0.54-1.61); .796	1.02 (0.59-1.78); .939
First contact: speech/learning vs pediatrician	-	-	-	**2.50 (1.34-4.66); .004**	**2.27 (1.20-4.27); .011**

Predictor	M1	M2	M3	M4	M5
First contact: other vs pediatrician	-	-	-	**2.90 (1.30-6.51); .010**	**2.72 (1.22-6.09); .015**
Prior specialist evaluation	-	-	-	0.88 (0.54-1.43); .595	0.76 (0.47-1.25); .288
Appointment difficulty: Sometimes vs No	-	-	-	-	1.41 (0.82-2.40); .212
Appointment difficulty: Yes vs No	-	-	-	-	**1.81 (1.08-3.02); .023**
Previous professional reassurance	-	-	-	-	**2.18 (1.40-3.38); <.001**

Footnotes: Cells show AOR (95% CI); p value. All models used the same complete-case sample (N = 299). M1 = background characteristics; M2 = M1 + recognition variables; M3 = M2 + caregiver response; M4 = M3 + help-seeking route; M5 = M4 + access and professional response. Bold indicates p < .05. AOR = adjusted odds ratio; CI = confidence interval.

Supplementary Table S4. Partial Proportional-Odds Generalized Ordered Logistic Regression Sensitivity Analysis (N = 299).

Panel A. Predictors satisfying the proportional-odds assumption

For these predictors, a single adjusted odds ratio applies across the cumulative response-category groupings.

Predictor	AOR (95% CI)	p
**Background characteristics**		
Age of individual with ASD, per year	1.03 (0.98-1.08)	.187
Male sex	1.63 (0.95-2.77)	.074
Maternal education, per category	0.95 (0.78-1.16)	.619
Household income, per category	1.15 (0.85-1.55)	.376
Urban residence	0.74 (0.46-1.19)	.212
**Recognition**		
Symptom-domain count, per domain	0.94 (0.83-1.07)	.367
**Caregiver response**		
Early signs initially not acted upon: Yes vs No	**2.34 (1.45-3.77)**	**<.001**
**Help-seeking route**		
General practitioner vs pediatrician	1.33 (0.70-2.52)	.391
Autism center vs pediatrician	1.03 (0.59-1.80)	.914
Speech/learning center vs pediatrician	**2.34 (1.23-4.43)**	**.009**
Other service vs pediatrician	**3.05 (1.35-6.91)**	**.007**
Prior specialist evaluation	0.74 (0.45-1.21)	.231
**Access and professional response**		
Appointment difficulty: Sometimes vs No	1.45 (0.85-2.48)	.178
Appointment difficulty: Yes vs No	**1.82 (1.08-3.06)**	**.023**
Previous professional reassurance	**2.16 (1.39-3.37)**	**<.001**

Panel B. Predictors allowed to vary across cumulative response-category groupings

For these predictors, the proportional-odds assumption was relaxed and grouping-specific adjusted odds ratios are reported.

Predictor	Cumulative response-category grouping	AOR (95% CI)	p
Age at first concern, per year	3–6 Months or longer	0.83 (0.65-1.06)	.130
	6 Months to 1 Year or longer	0.91 (0.70-1.18)	.474
	1–2 Years or longer	1.00 (0.77-1.30)	.992

Predictor	Cumulative response-category grouping	AOR (95% CI)	p
	More than 2 Years	1.17 (0.89-1.55)	.262
Early signs initially not acted upon: Maybe vs No	3–6 Months or longer	0.80 (0.35-1.81)	.587
	6 Months to 1 Year or longer	1.26 (0.56-2.85)	.574
	1–2 Years or longer	1.53 (0.67-3.48)	.309
	More than 2 Years	**2.61 (1.06-6.41)**	**.036**

Footnotes: AOR = adjusted odds ratio; CI = confidence interval; ASD = autism spectrum disorder. The partial proportional-odds generalized ordered logistic model used the same complete-case sample (N = 299) and covariate structure as the fully adjusted primary ordinal model (Model 5). Predictors without evidence of non-proportionality were constrained to a single proportional effect across cumulative response-category groupings and are presented in Panel A. Age at first concern and the Maybe-versus-No caregiver-response contrast showed evidence of non-proportionality and were therefore allowed to vary across cumulative response-category groupings; their grouping-specific estimates are presented in Panel B. Pediatrician and No were reference categories as applicable; female sex and non-urban residence were reference categories for binary predictors. Maternal education and household income were modeled per increasing category. AORs greater than 1 indicate greater odds of being in a longer diagnostic-delay category or being in the specified cumulative response-category grouping. Bold indicates $p < .05$. The partial proportional-odds model improved fit compared with the fully constrained proportional-odds model (LR $\chi^2 = 16.15$, df = 6, $p = .013$).

Supplementary Table S5. Sensitivity Analysis Replacing Symptom-Domain Count With Individual Reported Symptom Domains (N = 299).

Panel A. Individual Symptom Domains in the Fully Adjusted Model (M5)

Symptom domain	AOR (95% CI)	p
Speech delay	1.51 (0.77-2.95)	.229
Eye-contact difficulty	0.95 (0.56-1.60)	.838
Behavioral problems	1.09 (0.66-1.80)	.743
Social-interaction difficulty	0.97 (0.56-1.66)	.904
Repetitive behaviors	0.64 (0.39-1.06)	.081
Cognitive/developmental delay	0.87 (0.53-1.41)	.561

Panel B. Highlighted Coefficient-Level Findings: Primary M5 Versus Individual-Symptom M5

Predictor	Primary M5	Individual-symptom M5
**Caregiver response: Yes vs No**	**2.21 (1.38-3.54); p=.001**	**2.25 (1.39-3.62); p<.001**
**Speech/learning center vs pediatrician**	**2.27 (1.20-4.27); p=.011**	**2.20 (1.17-4.16); p=.015**
**Other service vs pediatrician**	**2.72 (1.22-6.09); p=.015**	**2.71 (1.19-6.14); p=.017**
**Appointment difficulty: Yes vs No**	**1.81 (1.08-3.02); p=.023**	**1.76 (1.05-2.96); p=.031**
**Previous professional reassurance**	**2.18 (1.40-3.38); p<.001**	**2.20 (1.40-3.44); p<.001**

Panel C. Speech-Delay Estimate Across the Sequential Sensitivity Models

Model	Speech-delay AOR (95% CI)	p
**M2**	**1.95 (1.02-3.72)**	**.042**
M3	1.91 (1.00-3.65)	.051
M4	1.64 (0.85-3.19)	.142
M5	1.51 (0.77-2.95)	.229

Footnotes: AOR = adjusted odds ratio; CI = confidence interval. The sensitivity models used the same complete-case sample and sequential covariate structure as the primary M1-M5 analysis, except that symptom-domain count was replaced by the six individual symptom indicators. The individual-symptom M5 did not significantly improve fit compared with the primary symptom-domain-count M5 (LR $\chi^2 = 4.20$, df = 5, $p = .521$). Bold indicates $p < .05$.

Supplementary Table S6. Sensitivity Analyses Accounting for Explicit Unknown/Do Not Remember Responses.

Panel A. Selected Associations From the Multivariable Known-Versus-Unknown Timing Model (N = 379)

Predictor	AOR (95% CI)	p
**Unable to recall number of evaluations**	**2.70 (1.26-5.78)**	**.011**
**Unknown previous professional reassurance**	**3.96 (1.19-13.23)**	**.025**

Panel B. Highlighted Coefficient-Level Findings: Primary M5 Versus Unknown-Inclusive M5

Predictor	Primary M5	Unknown-inclusive M5
**Caregiver response: Yes vs No**	**2.21; p=.001**	**2.14; p=.001**
**Speech/learning center vs pediatrician**	**2.27; p=.011**	**2.29; p=.008**
**Other service vs pediatrician**	**2.72; p=.015**	**2.55; p=.015**
**Appointment difficulty: Yes vs No**	**1.81; p=.023**	**1.79; p=.022**
**Previous professional reassurance: Yes vs No**	**2.18; p<.001**	**2.17; p<.001**

Panel C. Explicit Unknown Categories in the Unknown-Inclusive M5

Unknown category	AOR (95% CI)	p
Prior specialist evaluation: Unknown vs No	3.28 (0.97-11.11)	.057
**Appointment difficulty: Unknown vs No**	**4.41 (1.17-16.69)**	**.029**
Professional reassurance: Unknown vs No	2.40 (0.89-6.46)	.084

Footnotes: AOR = adjusted odds ratio; CI = confidence interval. "Do not remember" diagnostic timing was not treated as an ordinal delay category because it has no ordered position relative to the five delay categories. Timing availability was modeled separately as known versus unknown. The known-versus-unknown model included N = 379 participants with the required covariates (334 known, 45 unknown). The Unknown-inclusive ordinal sensitivity model included N = 331 participants with known diagnostic timing. Explicit Unknown predictor responses were retained as separate categories. Associations involving small Unknown subgroups were interpreted cautiously.
